# Characterization and Emulsifying Ability of Cassava Peels Solubilized Using Hydrothermal Treatments

**DOI:** 10.3390/polym17040496

**Published:** 2025-02-13

**Authors:** Jane Chizie Ogbonna, Mitsutoshi Nakajima, Marcos Antonio das Neves

**Affiliations:** 1 Doctoral Program in Life Science Innovation, Graduate School of Science and Technology, University of Tsukuba, Tsukuba 305-8577, Japan; s2230323@s.tsukuba.ac.jp; 2 Alliance for Research on the Mediterranean and North Africa (ARENA), University of Tsukuba, Tsukuba 305-8572, Japan; nakajima.m.fu@u.tsukuba.ac.jp; 3 Institute of Life and Environmental Sciences, University of Tsukuba, Tsukuba 305-8577, Japan

**Keywords:** cassava peel, subcritical water, extraction, emulsifier, co-emulsifier, interfacial tension, sustainable food technology

## Abstract

Cassava peels are rich in polysaccharides but highly unexplored and underutilized, as they could be used to meet the increasing demand for clean-label foods. This study investigated the effect of temperature on the solubilization of cassava peel during hydrothermal treatment to determine the emulsifying ability of solubilized cassava peel (SCP). Subcritical water conditions were employed via hydrothermal (120–200 °C; 2 MPa) or autoclave (127 °C; 0.2 MPa) treatments to solubilize cassava peels. The composition of the SCPs was determined, and their emulsifying ability was assessed using interfacial tension and zeta potential measurements. Under the best treatment conditions (140 °C at 2 MPa [hydrothermal]; 127 °C at 0.2 MPa [autoclave]), SCPs reduced interfacial tension against soybean oil to 12.9 mN/m and 13.4 mN/m, respectively. A strengthened co-emulsifier system was developed by incorporating SCPs with *Quillaja* saponins (QS) or Tween 20 to enhance the performance. Dynamic interfacial tension and zeta potential measurements revealed synergistic interactions, showing a remarkable reduction in interfacial tension from 12.94 to 5.33 mN/m. This suggests that the SCP has a surfactant-like structure owing to its amphiphilic structure and hydrophobic chains (nonpolar region) attached to the -OH functional group (polar region). Combining a second surface-active compound or co-emulsifier results in an additive effect, reducing the interfacial tension. These findings provide novel insights into carbohydrate-saponin binding and elucidate the impact of peel composition, concentration, and hydrothermal treatment conditions on co-emulsifier system performance, which will assist in the development of emulsifiers, contributing to the advancement of clean-label food technologies, effectively replacing synthetic emulsifiers in food formulations, and offering both sustainability and functionality. A systematic investigation of processing conditions and co-emulsifier interactions provides a practical framework for developing high-performance natural emulsifiers from agricultural waste.

## 1. Introduction

Food wastes, including agricultural by-products, present a significant global challenge with economic, environmental, and social implications. In 2023, the United States alone generated 60.3 billion kg of food waste, whereas Japan discards approximately 18 billion kg annually, equivalent to 40% of its national food production [1,2]. Developed countries produce more food waste than developing nations [3,4]. Among the various types of food waste, cassava processing generates substantial amounts of peel, often discarded and underutilized. Cassava (*Manihot esculenta* Crantz) is a staple crop in several tropical and subtropical regions. Global cassava production reached 30.3 billion kg in 2020, with Nigeria being the largest producer [5]. Cassava peel, which constitutes approximately 15–20% of the root weight, is rich in starch (50–70% dry weight) and dietary fiber (10–30% dry weight) and is a potentially valuable resource [6,7]. However, these peels are often considered waste contributing to environmental pollution when not properly managed [8].

The increasing focus on sustainable polymer science has led to extensive research on modifying and functionalizing natural polysaccharides obtained from agricultural byproducts. Cassava peels, rich in starch and cellulose polymers, are an abundant source of natural polysaccharides that can be modified for various applications through controlled depolymerization. The transformation of complex polysaccharides into functional materials addresses the growing need for sustainable polymer-based solutions. Hydrothermal treatment (HTS) is a process that uses hot water as the primary solvent, which eliminates the need for organic solvents and reduces environmental impact [9]. They can also be used to process wet biomass, reduce energy requirements for feedstock drying [10], and recover valuable compounds in the liquid phase, including nutrients and bioactive substances [11]. HTS uses water under subcritical conditions (120–370 °C at 0.2–25 MPa) as a solvent and a reactant, exploiting water’s unique properties under these conditions [12]. Under subcritical conditions, water exhibits decreased density and dielectric constant, increased hydrocarbon solubility, and enhanced ionic production of H^+^ and OH^−^ [12,13]. This makes Hydrothermal treatment (HTS) a sustainable processing method for cassava peel, as it neither generates additional waste streams nor requires chemical additives. These properties enable the efficient breakdown of the lignocellulosic structure of cassava peels without the need for acid/base catalysts or subsequent neutralization steps. However, autoclave treatment can be employed to compare the effects of temperature and pressure for a lower-cost and more sustainable process. A previous study compared the energy efficiencies of autoclaves and conventional HTS reactors [14]. Despite several studies employing HTS, it has seldom been used independently as a pretreatment for enzymatic hydrolysis. Recent studies have demonstrated the potential of HTS for valorizing cassava peels. Bayitse et al. [15] optimized the enzymatic hydrolysis conditions for cassava peel, achieving up to 98% hydrolysis efficiency and producing substantial amounts of fermentable sugars. Similarly, Olanbiwoninu and Odunfa [16] highlighted the importance of pretreatment methods, including hydrothermal processes, for enhancing the bioconversion of cassava peels into fermentable sugars.

Hydrothermal treatment is crucial in detoxifying cassava peels containing cyanogenic glycosides that can be harmful if consumed directly. Olafadehan et al. [17] demonstrated that processing methods, including hydrothermal treatment, could effectively reduce hydrogen cyanide concentrations in cassava peels. According to the European Food Safety Authority (EFSA), the safety limit for cyanide in cassava products is 2–10 mg/kg. This detoxification process is essential for utilizing cassava peels in livestock feed because untreated peels can pose health risks owing to their cyanogenic content. Moreover, the hydrothermal carbonization of cassava peel has been explored as a method for producing hydrochar. This is a carbon-rich material that can be used as a soil amendment or adsorbent for water treatment applications. Pratiwi et al. [18] successfully synthesized hydrochar from cassava peel using hydrothermal carbonization, demonstrating its potential as a hard-water softener [18]. This dual application of cassava peel, both as feedstock for bioenergy and as a material for environmental remediation, highlights the versatility of hydrothermal treatment in waste valorization, which aligns with the principles of circular economy and biorefinery concepts, potentially transforming cassava waste into valuable products [17].

Subcritical water treatment of cassava peel concentrates and preserves bioactive compounds, including polysaccharides and lignocellulosic components, contributing to their emulsifying properties. These compounds can stabilize emulsions by providing a network that traps oil droplets while improving the digestibility of complex carbohydrates through partial hydrolysis [19,20]. Although synthetic emulsifiers currently dominate 70% of the market due to their cost-effectiveness and availability, their production from primary agricultural resources may compromise food security and sustainable development goals. Therefore, the valorization of plant byproducts presents a more sustainable alternative for emulsifier production, aligning with the need for sustainable practices in the food industry [21,22,23]. Despite the abundance of polysaccharides in cassava peels, few studies have been conducted on their emulsifying properties. This study aimed to fill this gap by exploring the potential of cassava peel for emulsion stabilization, highlighting its promising role in the food industry.

However, cassava peel is a complex matrix of several compounds, and reducing the isolation and purification of a single compound can lead to lower costs. A study by Sulaeman et al. [19] showed the zeta potentials of cassava peels after microwave processing at 120 °C to be between 0.8 and 1.2 mV, which is not a desirable trait for emulsion stabilization. Hence, this study further explored the introduction of other emulsifiers with greater surface activity to strengthen the potential of cassava peel and to form synergistic interactions for more desirable emulsion properties.

*Quillaja* saponin is a plant-based, nonionic, and commercially available emulsifier, whereas polyoxyethylene sorbitan monolaurate, commercially known as Tween 20, is a synthetic nonionic emulsifier. *Quillaja* saponins sourced from *Quillaja* trees are preferred over other natural/plant-based emulsifiers because of their low molecular weights. They consist of polar sugar moieties attached to a nonpolar triterpene or steroid backbone, making these amphiphilic molecules highly active on their surfaces [24] and exhibit excellent techno-functional properties. However, the high cost of *Quillaja* significantly hinders its use in most commercial applications. In addition, the issues of sustainable farming and sourcing to meet the demand must be addressed [25]. Tween 20 is widely used because of its availability, low cost, and efficient emulsifying properties [26]. It has been approved as a non-toxic synthetic emulsifier by several regulatory bodies, such as the US Food and Drug Administration (FDA) and European Food Safety Authority (EFSA).

The present study addresses critical gaps in sustainable food technology through an innovative approach to cassava peel valorization. While previous research has explored various aspects of cassava waste management, this work pioneers the systematic investigation of hydrothermal processing for natural emulsifier development. Specifically, this study aims to (1) develop and optimize a chemical-free, subcritical water process for transforming cassava peels into functional emulsifiers; (2) establish the relationship between processing parameters and emulsifying properties; and (3) create a novel co-emulsifier system with *Quillaja* saponins to enhance stability while maintaining clean-label status. This research is particularly significant given the current dominance of synthetic emulsifiers (70% market share) and the massive scale of cassava waste generation (15–20% of 303 billion kg produced globally in 2020). By systematically investigating the effects of temperature, pressure, and residence time on extract properties, this study seeks to demonstrate how hydrothermal processing can be precisely controlled to generate customized emulsifiers for food, cosmetic, and pharmaceutical applications. This approach addresses the pressing need for clean-label ingredients and establishes a new paradigm for sustainable waste valorization, contributing to both circular economy principles and the advancement of green chemistry in food technology.

## 2. Materials and Methods

### 2.1. Raw Materials and Sample Preparation

Fresh cassava tubers (variety TMS 98/2101) were obtained from Nigerian Starch Mills Ltd. (Iheala, Anambra State, Nigeria). The tubers were thoroughly washed under clean, running tap water to remove dirt and debris. The tubers were manually peeled. The peels were sun-dried to a constant weight in the month of February when the average temperature and humidity were approximately 36 °C with 10% moisture content and collected for further processing. The cassava peels were milled using a rasper mill and sundried during the warmer months in Nigeria, in February, when the average temperature was approximately 36 °C to a constant weight with 10% moisture content, and the samples were dried to constant weight in an oven at 105 °C (ADVANTEC DRM620DB, Saito, Japan) to ensure consistency across all procedures. Once the cassava peels were adequately dried, they were sealed in airtight bags to prevent moisture absorption and to maintain sample quality. The sealed bags were stored in a cool, dry place before being transported to the laboratory for further analysis. The total carbohydrate, protein, ash, and cyanide contents of raw and dried cassava peels were determined.

### 2.2. Hydrothermal Treatment

#### 2.2.1. Hydrothermal Treatment of Cassava Peel Powder

**Figure 1:**
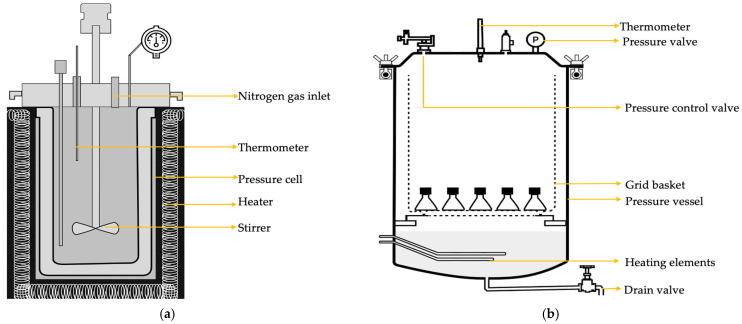
Schematic diagram of the equipment used for hydrothermal treatment: (**a**) Hydrothermal treatment (HTS) reactor and (**b**) autoclave.

The cassava peel powder was solubilized in a single-batch hydrothermal treatment (HTS) reactor, as shown in Figure 1a. Two hundred grams of a suspension containing 10% (wt) of powdered cassava peels dispersed in ultrapure water (18.2 MΩ·cm at 25 °C, obtained from a Allium comfort II system, Sartorius AG, Göttingen, Germany) was placed in a 250 mL stainless steel pressure cell. The suspension was mixed using a magnetic stirrer at 500 rpm for 30 min at room temperature (23 ± 2 °C), then the pressure cell was placed in the HTS reactor and sealed. The outer heating jacket was positioned, the internal stirrer was turned on at 100 rpm for homogeneity during the treatment, and the chamber was pressurized using nitrogen gas. All operating conditions were monitored and controlled during hydrothermal treatment, using a control panel.

Before each experiment, a cold-pressure test was conducted to verify the system’s integrity. Nitrogen gas (99.9% purity) was introduced into the sealed reactor until a pressure of 2.0 MPa was reached. The pressure was monitored for 10 min to ensure that there were no leakages and that the pressure distribution was uniform throughout the system. Different temperatures of 120, 140, 160, 180, or 200 °C were exerted with a pressure of 2 ± 0.2 MPa to determine the most suitable. The residence time was set as 15 min.

#### 2.2.2. Separation of Solubilized Cassava Peels (SCPs)

Following the hydrothermal treatment, a two-step separation process was used to separate the solubilized fraction from the solid residue. The temperature was maintained at 25 °C during separation. First, the solubilized contents were transferred to four 50 mL centrifuge tubes and centrifuged at 10,000× *g* for 30 min at 25 °C. This step effectively separated the bulk of the insoluble solid residue from the solubilized liquid fraction.

Next, the supernatant was carefully decanted and subjected to vacuum filtration (vacuum pump MDA-015, ULVAC Kiko, Inc., Saito, Japan) at pressure 0.1 ± 0.02 MPa while using a glass microfiber filter paper (pore size, 1.2 μm; Whatman GFC, Buckinghamshire, UK), to remove any remaining fine particles. The filtrate (hereafter referred to as SCPs) was collected in amber glass bottles and stored at 4 °C until further analysis. One portion of the SCPs was immediately frozen at −80 °C, and the remaining portions were freeze-dried for morphological analysis. The solid residue retained on the filter paper was carefully recovered, combined with the centrifuge pellet, and dried in an oven at 105 °C for 24 h to determine the dry weight of the residual solids. All separations were performed in triplicates to ensure reproducibility.

### 2.3. Characterization of SCPs

#### 2.3.1. Solubilization Yield

Specific amounts of SCPs, obtained using either the HTS reactor or autoclave, were separately placed on aluminum pans and dried in an oven (ADVANTEC DRM620DB, Tokyo, Japan) at 105 °C for 6 h, or until constant weight. After weighing, the solubilization yield was calculated using Equation (1) [23]:

(1) $$Solubilization\;yield\%=\frac{Weight\;of\;Solubilized\;Cassava\;Peels\;(g)\;(on\;dry\;basis)}{Weight\;of\;untreated\;cassava\;peels\;(g)\;(on\;dry\;basis)}\times 100$$

#### 2.3.2. Chemical Composition of SCPs

The total carbohydrate content was determined using the phenol-sulfuric acid method [27]. A calibration curve was constructed using glucose as a standard. Working standard volumes (0, 0.2, 0.4, 0.6, 0.8, or 1 mL) were added to 12 mL tubes and made up to 1 mL with ultrapure water. One milliliter of a 5% phenol solution and 5 mL of 98% sulfuric acid were added to each tube and gently mixed. After 10 min, the tubes were placed in a 25 °C water bath for 20 min. Absorbance was measured at 490 nm using a UV–Vis spectrophotometer (JASCO Co., Hachioji, Japan).

The cyanide concentration was determined using Nagashima’s pyridine-pyrazolone method [28]. Samples of various concentrations (0, 20, 40, 60, 80, or 100%) were prepared in 50 mL volumetric flasks, each containing 20 mL of the sample. Four milliliters of buffer (pH 4.7) and 0.5 mL of chloramine-T solution were added to each flask. After mixing, the solutions were maintained at 25 ± 1 °C for 30 min. This was followed by the addition of 10 mL of the pyridine-pyrazolone solution, and the volume was made up to 50 mL with distilled water. The flasks were corked and maintained at 25 °C for another 30 min. The absorbance was measured at 620 nm using a UV-Vis spectrophotometer.

Crude protein content was determined by total nitrogen analysis using a Perkin–Elmer 2400 II CHNS elemental analyzer (Waltham, MA, USA). Nitrogen values were converted into protein estimates using a standard nitrogen-to-protein conversion factor of 6.25 [29].

To determine the ash content, a pre-weighed sample of the SCPs was first dried in a crucible at 105 °C for 6 h until its weight stabilized. The dried sample was then transferred to a muffle furnace (FO Yamato, Tokyo, Japan) and incinerated at 575 °C for 6 h, following the National Renewable Energy Laboratory (NREL) protocol [30]. Ash content was calculated by measuring the difference between the initial sample weight and the residue weight after incineration.

#### 2.3.3. Morphological Analysis of SCPs

The morphological characteristics of the SCPs obtained using either HTS or autoclave treatment were examined using scanning electron microscopy (SEM; TM-1000 Miniscope, Hitachi High Technologies, Tokyo, Japan) for high-resolution imaging of the sample surfaces. SCPs were initially freeze-dried at −80 °C to preserve their structural integrity. An aliquot of dry SCP (approximately 0.5 g) was carefully distributed onto a double-sided conductive carbon tape. To ensure uniform coverage and minimize charging effects, the powder was gently spread across the adhesive surface using a spatula. Excess particles were gently removed to prevent loose debris from interfering with the imaging. The chamber was evacuated to a high vacuum (approximately 10^−5^ Pa) to facilitate the electron beam operation, and the image was captured at 50× magnification.

### 2.4. Measurement of Surface Activity of SCPs

The surface activity of the SCPs obtained using either HTS or an autoclave was evaluated by measuring the zeta potential and interfacial tension between the SCPs and soybean oil [22].

Interfacial tension measurements were performed with an automated interfacial tensiometer (PD-W; Kyowa Interface Science Co., Ltd., Saitama, Japan) using the pendant-drop method. SCP samples were loaded into a 1 mL glass syringe, and soybean oil was placed in a glass cuvette underneath the syringe. A droplet was formed to its maximum volume immediately before dropping into oil, and its image was captured using a high-resolution camera. The interfacial tension was then automatically calculated using the Young-Laplace equation based on droplet size and shape parameters. This method allowed for precise quantification of the interfacial properties, providing insights into the surface-active behavior of the treated cassava peel components, such as starch, at the oil–water interface.

The zeta potentials of the SCP samples were determined using a zetasizer (Nano-ZS; Malvern Instruments Ltd., Worcestershire, UK). The samples were diluted 100 times with Milli-Q water to prevent multiple scattering effects.

### 2.5. Emulsifying Ability of SCPs

Initially, the formulation of oil-in-water emulsions was stabilized using only SCP as an emulsifier. It was used to investigate the emulsifying ability of SCPs obtained using HTS (140 °C at 2 MPa for 15 min). In addition, various ratios of SCPs and 1% (wt) *Quillaja* saponin were studied.

#### 2.5.1. Formulation of Oil-in-Water (O/W) Emulsion Stabilized by SCPs

SCPs obtained using an HTS reactor operated at 140 °C at 2 MPa for 15 min were used in the aqueous phase, whereas soybean oil was used in the disperse phase for emulsion preparation. Fifty milliliters of a pre-mixture containing 5% (wt) pure soybean oil dispersed in the SCP aqueous phase was prepared and homogenized using a rotor-stator homogenizer (Polytron PT-3000, Kinematica-AG, Littace, Switzerland) at 7000 rpm for 5 min to form coarse emulsions. Fine emulsions were obtained by passing the samples through a high-pressure homogenizer (NanoVater NV200; Yoshida Kikai, Nagoya, Japan) operated at 90 MPa for four consecutive cycles. The temperature during homogenization was maintained at 25 ± 1 °C

The storage stabilities of the prepared emulsions were assessed by monitoring their droplet sizes over time with samples collected on days 0, 3, 7, and 10. The emulsions were either stored at 5 °C or 25 °C for up to 10 days. The size distribution and mean droplet diameter (*d*_3,2_) of the emulsion droplets were measured using a static laser diffraction particle size analyzer (LS 13,320, Beckman Coulter, Brea, CA, USA) at regular intervals during storage. The refractive indices of the oil and aqueous phases are 1.467 and 1.330, respectively.

#### 2.5.2. Strengthening the Surface Activity and Emulsifying Ability of SCPs Using Co-Emulsifiers

To enhance the surface activity of SCPs obtained using HTS (140 °C at 2 MPa for 15 min), two commercial emulsifiers, *Quillaja* saponins and Tween 20, were used separately as co-emulsifiers.

Initially, aqueous solutions containing 1% (wt) of each co-emulsifier were mixed with SCPs at weight ratios of 10:0, 7.5:2.5, 5:5, 2.5:7.5, or 0:10 (where the first number indicates the weight ratio of the SCP, and the second number indicates the weight ratio of the respective co-emulsifier). Next, the interfacial tension between each co-emulsifier system and soybean oil was determined, as well as their respective zeta potential values (as described in the previous section).

The SCPs, *Quillaja* saponins, and Tween 20 surface charges were evaluated individually and as co-emulsifiers by measuring their respective zeta potentials.

#### 2.5.3. Formulation and Stability Evaluation of O/W Emulsions Prepared Using SCPs and *Quillaja* Saponin as Co-Emulsifiers

The emulsions were formulated as described in Section 2.5.1. The aqueous phase consisted of SCP and 1% (wt) *Quillaja* saponin in ultrapure water at various ratios: 10:0, 7.5:2.5, 5:5, 2.5:7.5, and 0:10 (weight ratio; SCPs: *Quillaja* saponin).

The storage stability was investigated by monitoring the droplet sizes of the emulsions stored either at 5 °C or 25 °C for 10 d. The mean droplet diameter (*d*_3,2_) was measured using a static laser diffraction particle size analyzer (LS 13,320, Beckman Coulter, Brea, CA, USA).

### 2.6. Statistical Analysis

All measurements were performed in triplicates for statistical analyses. The statistical test used was a one-way analysis of variance (ANOVA), while Tukey’s HSD was used for post hoc comparison. The analyses were performed using the SPSS statistics software (version 29.0; BM Corp., Armonk, NY, USA). Results are expressed as mean values ± standard deviation. Differences were considered statistically significant at *p* < 0.05.

## 3. Results and Discussion

### 3.1. Characterization of Untreated Cassava Peels

Table 1 shows the composition of the sun-dried cassava peels. A notable carbohydrate content of 38.5% (wt) was observed, which aligns with previous studies that reported carbohydrate levels in cassava peel ranging from 35% to 80% [31]. The ash content of 8.6% (wt) was slightly higher than the 5% reported by Adekunle et al. [32]. The total protein content of 9.3% (wt) was slightly higher than the typical values of 4.8–6.5% observed in a study by Lukuyu et al. [33], suggesting differences in cassava variety and/or effects of the processing methods. The cyanide concentration (6.5 μg/g) was relatively low compared to fresh cassava peels, which can contain up to 1000 mg/kg of cyanogenic compounds and well below safe limits of 2–10 mg/kg according to EFSA [34], indicating effective detoxification through sun-drying. These findings contribute to the growing knowledge of cassava peel composition and underscore the potential of sun-dried cassava peel as a valuable resource for various applications, including emulsion stabilization.

### 3.2. Composition of Cassava Peels Solubilized Using an Autoclave

Table 1 also presents the composition of cassava peels solubilized using an autoclave at 127 °C and 0.2 MPa. In addition, the solubilization yield was 25.98%, indicating a relatively efficient extraction process, likely due to the high temperature breaking down the cell wall structures, as previously reported by Ubalua [35]. The total carbohydrate content of 3.34% (wt.) was lower than expected, possibly because of the hydrolysis of complex carbohydrates during processing. The ash content of 3.89% (wt) fell within the typical range for cassava peels (4–10%) reported by Kayiwa et al. [36], representing the mineral content of the solubilized fraction.

The total protein content (0.765% [wt]) was relatively low (Bayata [37]) probably due to protein insolubility under the given conditions. Notably, the cyanide content (4.33 μg/g) was relatively low, indicating that the autoclave treatment was effective in reducing this potentially toxic compound, aligning with Nambisan’s [38] findings on the efficacy of high-temperature treatments in reducing cyanogenic compounds. Overall, these results suggest that while autoclave treatment effectively solubilized a significant portion of cassava peels and reduced the cyanide content, further optimization may be required to increase the yield of desirable components, such as proteins or specific carbohydrates, for potential use as emulsifiers.

### 3.3. Operating Conditions for Treatment of Cassava Peels Using an HTS Reactor

**Figure 2:**
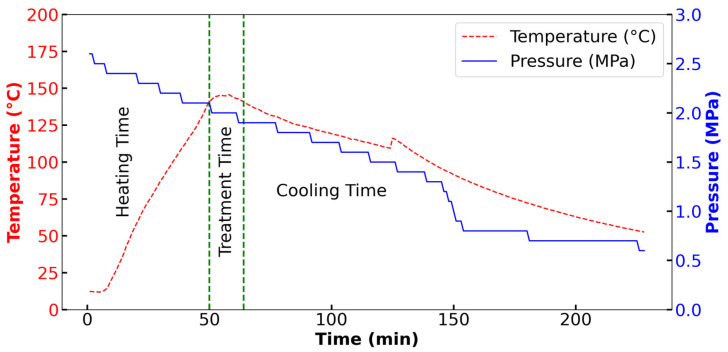
Temperature, pressure, and time profile of the hydrothermal solubilization reactor at 140 °C and 2 MPa with a holding time of 15 min.

Figure 2 shows the relationship between the temperature and pressure during the hydrothermal treatment. The following experimental parameters were used: treatment time of 15 min, pressure of 2 ± 0.4 MPa, and a target temperature of 140 ± 5 °C. The temperature jacket was preset to 500 °C to achieve rapid heating, enabling the system to reach the desired temperature (140 °C) in less than 50 min. The pressure was maintained at the target level from the onset of the experiment, owing to the initial pressurization. Upon reaching the target temperature, the 15 min treatment period commenced. At the end of the 15 min, the heating jacket was switched off, resulting in a rapid decrease in both temperature and pressure. The temperature profile exhibited an initial sharp increase, followed by a plateau during the holding time and a gradual decline. Concurrently, the pressure remained relatively constant during the heating and holding phases, followed by a steady decrease that accelerated towards the end of the process.

### 3.4. Solubilization Yield of SCPs and the Effect on pH

**Figure 3:**
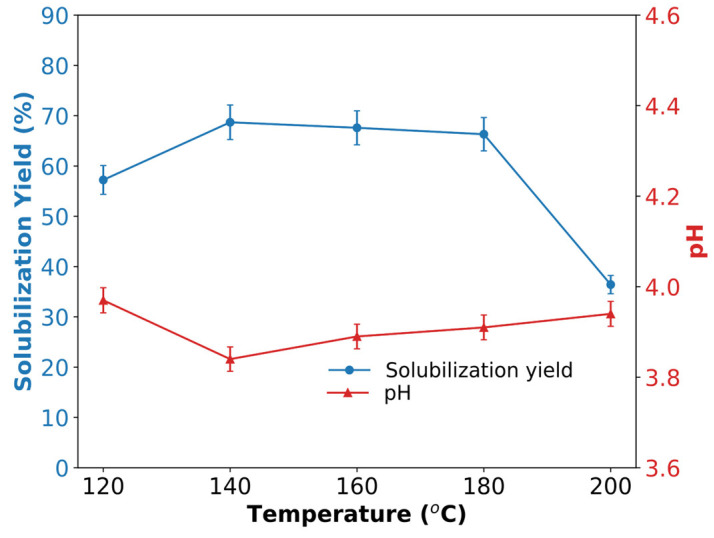
Effect of thermal treatment temperature on the solubilization yield and pH of solubilized cassava peels (SCPs) obtained using an HTS reactor.

The powdered cassava peels with a particle size of 445 ± 50 μm after various hydrothermal treatments were investigated. Figure 3 shows the temperature-dependent solubilization yield of cassava peels after HTS at 2 MPa and autoclave treatment at 0.2 MPa (Table 1) and the effect on pH. The HTS process demonstrates a bell-shaped yield curve, peaking at approximately 140–160 °C with yields approaching 70%, followed by a decline at higher temperatures. The highest yield was attained at the lowest pH, indicating a positive effect of acidic compounds on solubilization. This trend is consistent with the observed behavior of other lignocellulosic biomasses, such as corn stover and rice straw, after HTS [35]. The optimal temperature range likely corresponds to the effective breakdown of hemicellulose (150 °C to 180 °C) and partial depolymerization of cellulose (180 °C to 220 °C). The yield reduction at temperatures below 160 °C is due to the increased formation of solid residues (biochar) or gaseous products, as observed during material collection and in studies on HTS of woody biomass. The observed molecular structure and functionality changes demonstrate how controlled processing conditions can effectively modify natural polysaccharides to create functional polymeric materials with enhanced surface-active properties. The relationship between processing parameters and polymer characteristics provides insights into tailoring natural polysaccharides for specific applications.

Cassava peel primarily consists of starch (50–60%), cellulose (10–30%), hemicellulose (10–20%), and lignin (10–20%). Aromatic compounds from lignin decomposition may also contribute to the liquid yield at higher temperatures. The yield difference between HTS (57% at 120 °C) and autoclave treatment (26% at 127 °C) highlights the crucial role of pressure in enhancing hydrolysis reactions and maintaining water in a subcritical state, thereby improving mass transfer and solubility of biomass components. Sulaeman et al. [19] obtained a 45% yield of micro-fibrillated cellulose from cassava peels after microwave treatment at 120 °C but a decline in yield to 31% and 15% was observed at 170 °C and 220 °C, respectively, which is consistent with the results of the present study.

The decline in yield at 200 °C could indicate the onset of repolymerization reactions or increased gasification, which are often observed in HTS of other agricultural residues, such as sugarcane bagasse [23] or wheat straw [39] at similar temperatures. This comparison suggests that although cassava peels respond well to HTS, careful temperature control is essential to maximize the liquid product yield and potentially tailor the composition of the resulting solubilized materials for specific applications.

### 3.5. The Composition of SCPs Obtained Using an HTS Reactor

**Figure 4:**
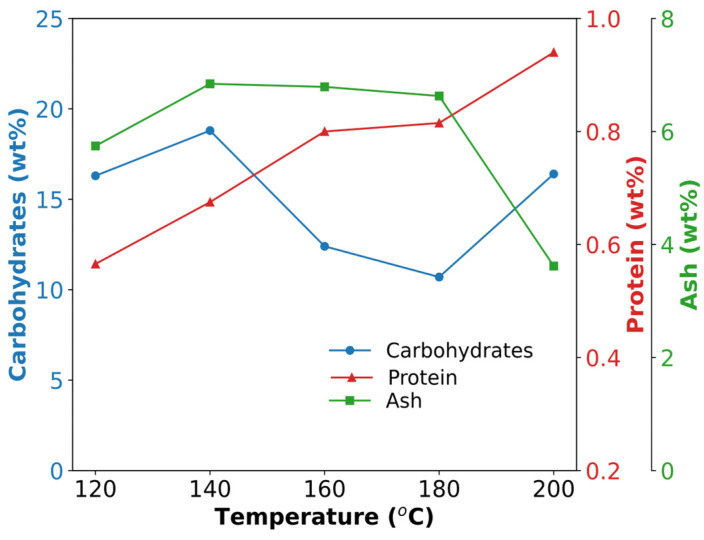
Effect of hydrothermal treatment temperature on the composition (carbohydrates, protein, and ash content) of SCPs obtained using an HTS reactor.

Figure 4 shows the total carbohydrates, proteins, and ash content of cassava peels under various hydrothermal treatment conditions of subcritical water extraction (120–200 °C at 2 MPa) and autoclave (127 °C at 0.2 MPa; Table 1). The changing properties of water can explain these trends under subcritical conditions and their effects on the biomolecules.

For carbohydrates, the yield increased during autoclave treatment (Table 1) at 120 °C, peaking at 140 °C, followed by a decline and another increase at 200 °C. This pattern likely reflected the initial breakdown of complex carbohydrates into simpler and more soluble forms with increasing temperature, followed by their potential degradation or transformation at higher temperatures. The increase at 200 °C could indicate further breakdown of resistant structures. The lowest yield was obtained under autoclave conditions at 3.3% (wt) because the lower pressure reduced material transfer.

Protein extraction showed a different trend, with a decrease in yield at 120 °C compared to autoclave conditions (0.8% (wt)), followed by a steady increase up to 200 °C. This aligns with previous studies on subcritical water extraction, in which higher temperatures enhanced the protein solubility and extraction efficiency [40]. This initial decrease may be due to protein denaturation or aggregation during the transition from autoclaving to subcritical conditions.

Ash content exhibited a similar pattern. It increased gradually from autoclave treatment to HTS at 140 °C, peaking at approximately 6% (wt), before steadily decreasing with a sharp decline at 200 °C. The initial increase in the ash content could be attributed to the concentration effect, as organic matter was solubilized and removed from the solid fraction. The subsequent decrease at higher temperatures may indicate the partial solubilization of inorganic compounds or their incorporation into the char formation.

The different trends between carbohydrates and proteins highlight the complex interplay between temperature, pressure, and water properties during subcritical water extraction. These findings are consistent with studies on other biomass sources, showing that optimizing the extraction conditions can selectively target different biomolecules [41,42]. These results suggest that careful control of the hydrothermal treatment conditions enables the extraction of targeted carbohydrates, ash, or proteins from cassava peels with potential improvement in the valorization of these agricultural byproducts.

**Figure 5:**
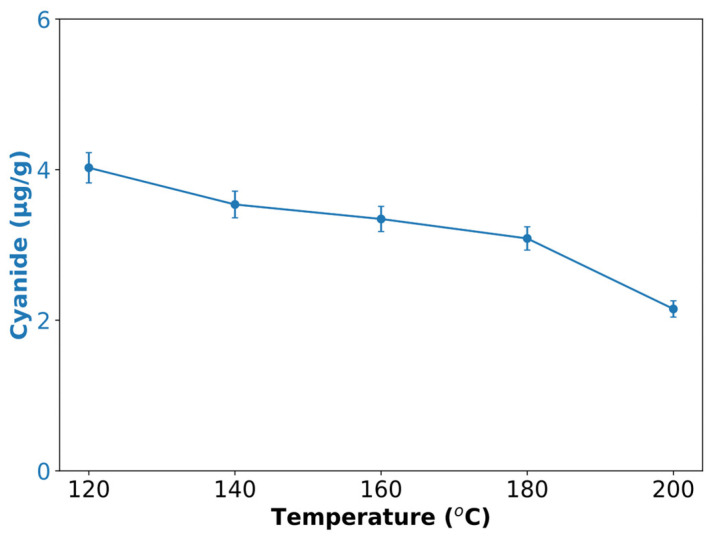
Effect of hydrothermal treatment temperature on cyanide content of SCPs obtained using an HTS reactor.

Figure 5 shows the effects of hydrothermal treatment on cyanide in cassava peels at various temperatures. A gradual decrease from autoclave treatment (127 °C at 0.2 MPa; Table 1) through increasing HTS temperature (120–200 °C at 2 MPa) was observed, starting at approximately 4.33 μg/g (autoclave) and declining to approximately 2.2 × 10^−4^ μg/g at 200 °C. This trend suggests thermal degradation of cyanogenic glycosides, particularly linamarin, which is prevalent in cassava peels. This decline aligns with studies on cassava detoxification, where heat treatment has been shown to reduce the cyanide content [43].

These results suggest that HTS effectively reduced the levels of toxic cyanogenic compounds, which is beneficial for various applications of treated biomass. The decrease in cyanide at higher temperatures (160 °C to 200 °C) implies further chemical changes and possibly the formation of volatile compounds.

Potential materials solubilized during this process include degraded products of starch, cellulose, and hemicellulose (e.g., glucose, xylose, and furfural), and phenolic compounds from lignin breakdown [35]. The reduction in CN content is particularly significant for cassava peel-derived products’ safety and potential applications.

The observed trends highlight the importance of temperature control in HTS for optimizing desired outcomes, such as cyanide reduction. These findings contribute to a broader understanding of HTS as a promising technology for valorizing agricultural wastes while addressing safety concerns associated with cyanogenic plants, such as cassava.

### 3.6. Morphological Characterization of Cassava Peels Before and After HTS

**Figure 6:**
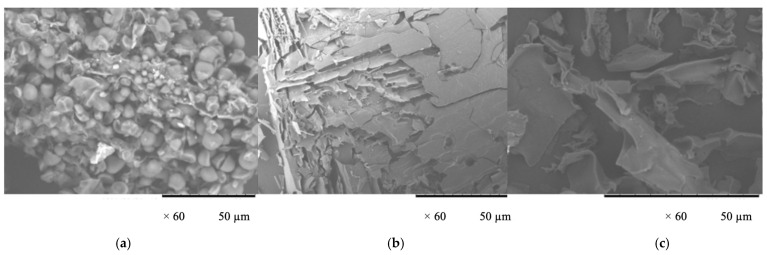
Scanning electron microscope (SEM) images of (**a**) untreated cassava peel powder; (**b**) cassava peels solubilized at 140 °C and 2 MPa (HTS reactor) and freeze-dried; and (**c**) cassava peels solubilized at 127 °C and 0.2 MPa (autoclave) and freeze-dried.

The SEM images in Figure 6 reveal distinct morphological changes in the cassava peels under different hydrothermal treatment conditions. Figure 6b shows cassava peels’ layered, flaky structure with significant surface disruption. This was likely due to subcritical water conditions causing the partial solubilization and restructuring of cell wall components, as observed in a similar study on biomass solubilization [41]. Figure 6c shows a less dramatic but noticeable alteration in the surface structure with visible fragmentation and opening of the cellular matrix. This aligns with the finding that autoclave treatment can cause the partial breakdown of plant cell walls [14]. In contrast, Figure 6a shows the untreated cassava peel powder, which exhibits a more compact, granular structure typical of native plant material. The progressive structural changes shown in Figure 6a–c demonstrate the increasing effectiveness of higher temperatures and pressures in breaking down the complex lignocellulosic structures of cassava peels. This breakdown is crucial for enhancing the accessibility and extractability of valuable components such as proteins and carbohydrates, as reported in studies on subcritical water extraction of biomass [12]. The observed morphological changes correlate with the increased extraction yields shown in the previous figures and highlight the importance of optimizing hydrothermal treatment conditions for efficient biomass valorization.

### 3.7. Interfacial Tension and Zeta Potentials of SCPs

Table 2 compares the interfacial tension (IT) and zeta potential of SCPs obtained using HTS at 140 °C and 2 MPa and autoclave treatment at 127 °C and 0.2 MPa.

The SCPs obtained using HTS showed a slightly higher interfacial tension (approximately 12.94 mN/m) than the autoclave-treated SCPs (approximately 12.84 mN/m). This marginal difference suggests that HTS at higher temperatures and pressures produces fewer surface-active compounds. The relatively low IT values for both treatments indicated the presence of surface-active molecules, including amphiphilic compounds derived from the breakdown of cell wall components and other biomolecules in the cassava peel.

The zeta potential values showed a more pronounced difference, with HTS-treated SCPs exhibiting a lower negative value (approximately −3 mV) than the autoclave-treated SCPs (approximately −8 mV). This difference indicated that HTS resulted in particles or colloidal systems with a reduced surface charge. The negative zeta potential in both cases suggested the presence of anionic groups on the surfaces of the extracted particles or molecules.

The observed differences between the HTS and autoclave treatments can be attributed to the more severe HTS conditions, which likely led to a more extensive breakdown of the biomass components. Potential materials solubilized during these processes include starch, cellulose, and hemicellulose, which yield various oligosaccharides and monosaccharides. In addition, lignin degradation products, such as phenolic compounds and organic acids, contribute to the surface-active properties and surface-charge characteristics of the extracts.

These findings align with studies on the HTS of other lignocellulosic biomasses, where the process conditions significantly influenced the physicochemical properties of the resulting bio-oils and aqueous fractions [40]. The lower magnitude of the zeta potential in the HTS-derived extracts may indicate reduced colloidal stability, which could have implications for potential applications in emulsion systems or as bio-based surfactants.

### 3.8. Characterization of O/W Emulsions Stabilized by SCPs

**Figure 7:**
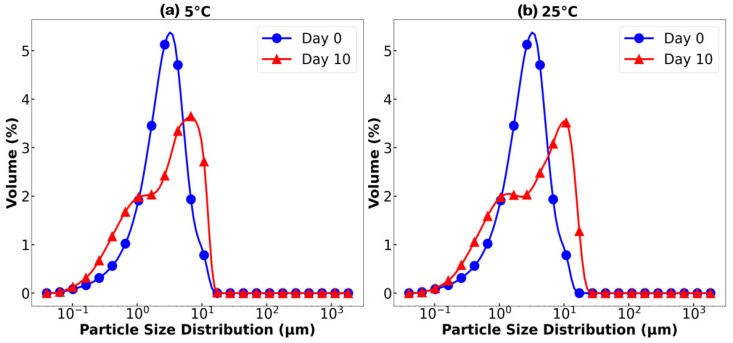
Droplet size distribution of oil-in-water (O/W) emulsions stabilized by SCPs and stored for 10 d at (**a**) 5 °C and (**b**) 25 °C.

**Figure 8:**
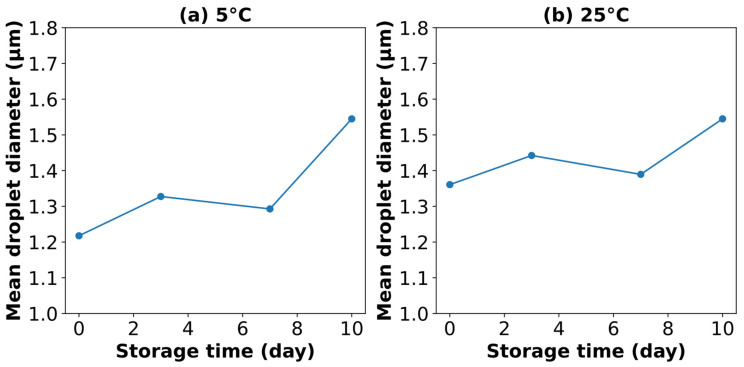
Mean droplet diameter (*d*_3,2_) of O/W emulsions stabilized by SCPs and stored for 10 d at (**a**) 5 °C and (**b**) 25 °C.

The emulsifying ability of SCPs obtained using HTS at 140 °C and 2 MPa was evaluated by analyzing the droplet size distribution and stability of O/W emulsions over a 10 d storage period at 5 °C and room temperature (25 °C). As shown in Figure 7, the particle size distribution exhibited a peak above 1 μm, indicating large droplet sizes, which are more prone to destabilization [44]. The mean droplet diameter, as shown in Figure 8, increased from day 0 to day 10, suggesting continuous coalescence and potential Ostwald ripening rather than improved stability [45]. This increase in droplet size, coupled with the bimodal distribution, indicates poor emulsion stability, likely due to insufficient surface activity, limited steric stabilization, and inadequate viscoelastic interfacial film formation by the SCP components [45,46].

The observed limitations in emulsion uniformity and stability over time when using SCPs alone clearly justify the need for co-emulsifiers to reduce interfacial tension, provide synergistic effects through complementary molecular structures, and optimize the overall hydrophilic–lipophilic balance of the emulsifier system [47,48]. These findings strongly support the exploration of co-emulsifier systems, such as saponins, to leverage the unique characteristics of cassava peel-derived components while addressing their inherent limitations in creating stable, uniform emulsions suitable for food and pharmaceutical applications. *Quillaja* saponins are promising candidates owing to their complementary amphiphilic structure, natural origin, excellent emulsifying properties, and potential synergistic interactions with the carbohydrate components of SCPs. Saponins can reduce the interfacial tension, provide effectual steric hindrance, and improve the pH and temperature stability of the emulsions. Combining SCP with saponins is hypothesized to create a more robust interfacial layer, potentially resulting in improved emulsion stability, smaller droplet sizes, and enhanced resistance to environmental stresses. This approach addresses the weaknesses observed in the emulsifying ability of SCPs, and aligns with the growing demand for natural clean-label emulsifiers in food and pharmaceutical applications. Further experimental studies comparing the performances of SCPs alone, saponins alone, and their combinations at various ratios are necessary to validate this hypothesis and optimize the co-emulsifier system for specific applications.

### 3.9. Surface Activity of SCPs

#### 3.9.1. Interfacial Tension of SCPs Obtained by Hydrothermal Treatment (HTS Reactor or Autoclave), Combined with Co-Emulsifiers

To enhance the emulsifying ability of solubilized cassava peels, they were blended with commercially available emulsifiers prior to formulation of O/W emulsions, and their surface activities were evaluated. This approach was used to increase electrostatic repulsion, one of the stabilizing mechanisms of effective emulsifiers, to mitigate coalescence and other destabilization phenomena. Two nonionic emulsifiers were selected for this study: *Quillaja* saponin, a plant-based compound derived from the bark of *Quillaja*, and Tween 20, a synthetic emulsifier. Both emulsifiers were used at various concentrations to evaluate their effects on the emulsion system. As the highest solubilization of cassava peels was obtained at 140 °C and 2 MPa in the HTS reactor, these conditions were applied for interfacial tension and zeta potential studies while comparing them with the least severe conditions using an autoclave at 127 °C and 0.2 MPa.

Comprehensive analyses of interfacial tension and zeta potential were conducted to evaluate the efficacy of these emulsifiers and their mixtures. These measurements provide insights into the ability of emulsifiers to reduce the O/W interface energy and assess the electrostatic repulsion between droplets, which are crucial factors for emulsion formation, stability, and long-term performance. Through these experimental investigations, this study aimed to elucidate the synergistic effects of combining different emulsifiers and optimizing the formulation to enhance emulsion stability and performance.

**Figure 9:**
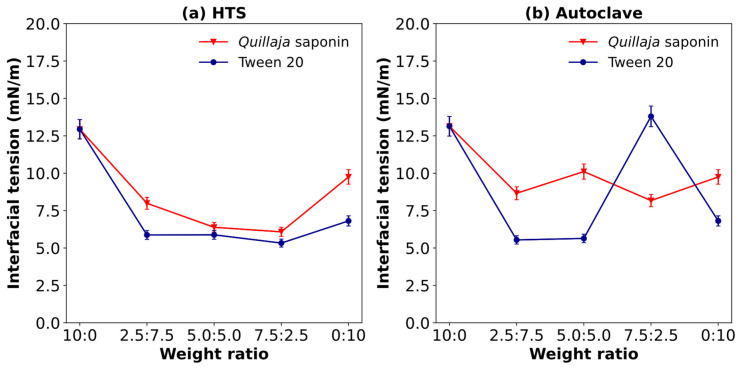
Effect of the weight ratio between SCPs and co-emulsifiers (*Quillaja* saponin or Tween 20) on interfacial tension: (**a**) 140 °C at 2 MPa (HTS reactor) and (**b**) 127 °C at 0.2 MPa (autoclave) (weight ratios of 10:0, 7.5:2.5, 5:5, 2.5:7.5, and 0:10 (SCP: co-emulsifier)).

Figure 9 shows the effects of various weight ratios of SCPs combined with *Quillaja* saponin or Tween 20 on interfacial tension. Pure *Quillaja* saponins exhibited higher interfacial tension (9.75 mN/m) than Tween 20 (6.81 mN/m), reflecting the structural differences between these emulsifiers. Tween 20, a synthetic nonionic surfactant, possesses a well-defined structure optimized for interfacial activity, while *Quillaja* extract contains diverse saponins with varying surface-active properties [49].

The addition of SCPs demonstrated distinct interaction patterns with both co-emulsifiers. The maximum reduction in interfacial tension occurred at 75% concentration, with *Quillaja* saponin decreasing from 9.75 to 6.08 mN/m and Tween 20 decreasing from 6.81 to 5.33 mN/m. This reduction suggests synergistic interactions between cassava peel components and the emulsifiers, likely resulting from complementary molecular structures, co-adsorption effects, and modified interfacial rheology [25].

The two co-emulsifiers displayed contrasting behaviors between mixing ratios of 7.5:2.5 and 2.5:7.5. *Quillaja* saponins showed increasing interfacial tension with higher SCP concentrations, while Tween 20 maintained relatively stable values. This stability indicates Tween 20’s superior compatibility with SCPs, attributed to its simpler molecular structure facilitating co-organization at the interface.

Interfacial tension values of other plant-derived emulsifiers typically exceed 10 mN/m when measured against soybean oil. Examples include solubilized sugarcane bagasse (14 mN/m) [23], *Gum arabica* (12.4 mN/m) [23], licorice root extract (10 mN/m) [49], argan shell extract (9.8–12 mN/m) [22], and *Limnophila aromatica* crude extract (12.58–16.18 mN/m) [50].

The effectiveness of cassava peel extract as a co-emulsifier demonstrates sensitivity to processing parameters. Under milder conditions (127 °C, 0.2 MPa), optimal performance occurred at different concentration ranges, suggesting the importance of calibrating processing conditions with extract concentration. This adaptability allows formulators to meet diverse processing requirements while maintaining performance standards.

From a sustainability perspective, this approach represents a significant advancement in waste valorization. Producing cassava peels under mild conditions (achievable in standard autoclaves) makes the technology more accessible to smaller-scale operations. Furthermore, the extract’s demonstrated effectiveness as a co-emulsifier presents opportunities for reducing synthetic emulsifier usage while creating value-added products from agricultural waste, which is particularly beneficial in cassava-producing regions [51,52,53].

#### 3.9.2. Zeta Potential of SCPs Obtained by Hydrothermal Treatment (HTS Reactor or Autoclave), Combined with Co-Emulsifiers

**Figure 10:**
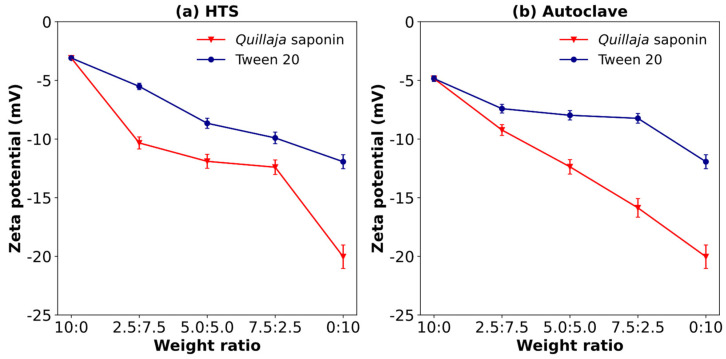
Effect of weight ratio of solubilized cassava peels (SCP) to co-emulsifiers (*Quillaja* saponin or Tween 20) on zeta potential: (**a**) 140 °C at 2 MPa (HTS reactor) and (**b**) 127 °C at 0.2 MPa (autoclave) (the weight ratios were 10:0, 7.5:2.5, 5:5, 2.5:7.5, and 0:10 (SCP: co-emulsifier)).

Figure 10a shows the effect of the weight ratio of cassava peel extracted at 140 °C and 2 MPa on the zeta potential of *Quillaja* saponins and Tween 20. This high-pressure treatment may enhance the extraction efficiency and potentially alter the chemical structure of the extracted compounds, thereby affecting their interactions with the emulsifiers at the interface. At a 0:10 ratio (pure co-emulsifier), there was a significant difference in the zeta potential between Tween 20 (−11.93 mV) and *Quillaja* saponins (−20.03 mV), indicating the superior electrostatic stabilization potential of *Quillaja* saponins in pure form. The introduction of SCPs resulted in distinct trends for each emulsifier. Tween 20 exhibits a non-monotonic trend, with a slight decrease to −9.90 mV at 7.5:2.5 and −8.66 mV at 5.0:5.0 and a notable increase in magnitude to −5.51 mV at 2.5:7.5. In contrast, *Quillaja* saponins show a consistent decrease in zeta potential magnitude from −12.40 mV at a ratio of 2.5:7.5 to −10.33 mV at a ratio of 7.5:2.5, which could be attributed to charge neutralization or screening effects between *Quillaja* saponins and cassava peel components.

At a ratio of 10:0, the SCP had a value of −3.09 mV, indicating that SCP dominates interfacial properties, though this low magnitude suggests limited electrostatic stability for emulsions formed solely with the extract. These trends revealed complex interactions between the extract and primary emulsifiers, with the optimal concentration for enhanced stability being emulsifier-dependent: *Quillaja* saponins showed better stability at lower SCP concentrations, while Tween 20 exhibited a potential synergistic effect at higher SCP concentrations. This complexity underscores the importance of carefully optimizing the SCP-to-emulsifier ratio in formulations to achieve the desired emulsion stability. 

Figure 10b shows the effect of weight ratio t of cassava peel solubilized at 127 °C and 0.2 MPa on the zeta potential of *Quillaja* saponins and Tween 20. It provides insight into the electrostatic stability of emulsions under milder processing conditions compared to that shown in Figure 7a. As the SCPs were introduced, complex and non-monotonic trends were observed in the zeta potential of both emulsifiers. For Tween 20, the zeta potential decreased from −11.93 mV at 0:10 to −7.97 mV at 2.5:7.5 and −7.41 mV at 5.0:5.0 before reaching its lowest value of −4.85 mV at 7.5:2.5. This nonlinear behavior suggests complex interactions between Tween 20 and the SCPs, possibly involving competitive adsorption or formation of mixed interfacial layers. A different pattern was observed for *Quillaja* saponins with SCPs. The zeta potential magnitude decreased sharply from −20.03 mV (0:10 ratio) to −13.1 mV (2.5:7.5 ratio), then further to −9.07 mV (5.0:5.0 ratio) and −9.40 mV (2.5:7.5 ratio), before dropping dramatically to −1.46 mV at a ratio of 10:0. This trend indicates progressive neutralization of the negative charge imparted by *Quillaja* saponins, possibly due to interactions with oppositely charged or neutral components of the cassava peel extract.

### 3.10. Characterization of O/W Emulsions Stabilized by SCPs Mixed with Quillaja Saponin as a Co-Emulsifier

**Figure 11:**
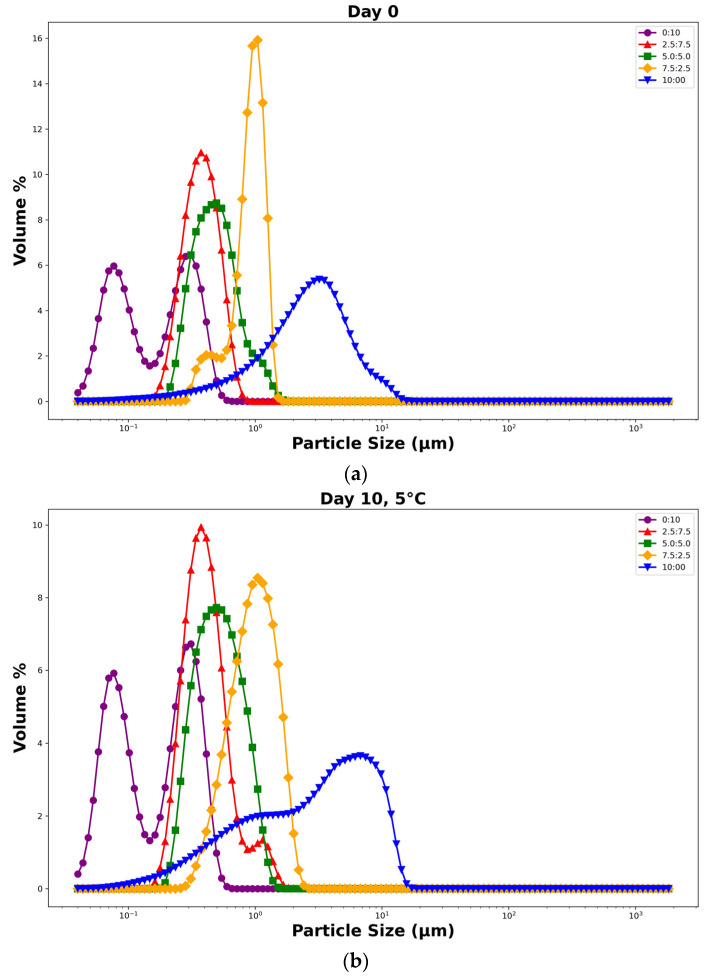

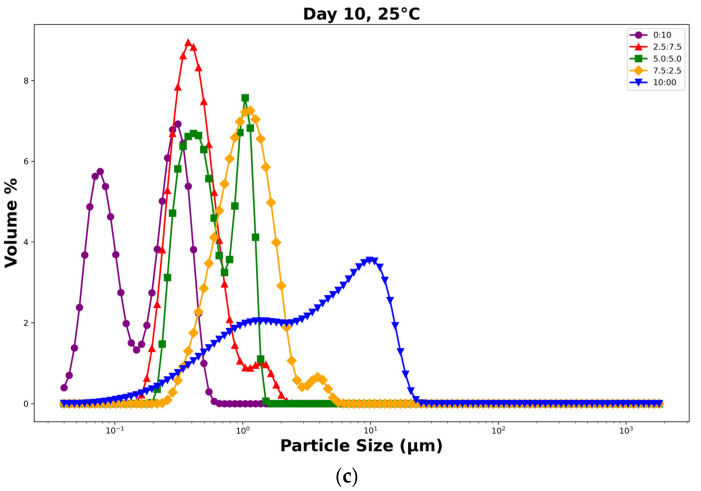
Droplet size distribution of O/W emulsions stabilized by SCPs or *Quillaja* saponins at various weight ratios, and stored for 10 d: (**a**) Fresh emulsion (day 0); (**b**) stored at 5 °C for 10 days; and (**c**) stored at 25 °C for 10 days (the weight ratios were 10:0, 7.5:2.5, 5:5, 2.5:7.5, and 0:10 (SCP: co-emulsifier)).

**Figure 12:**
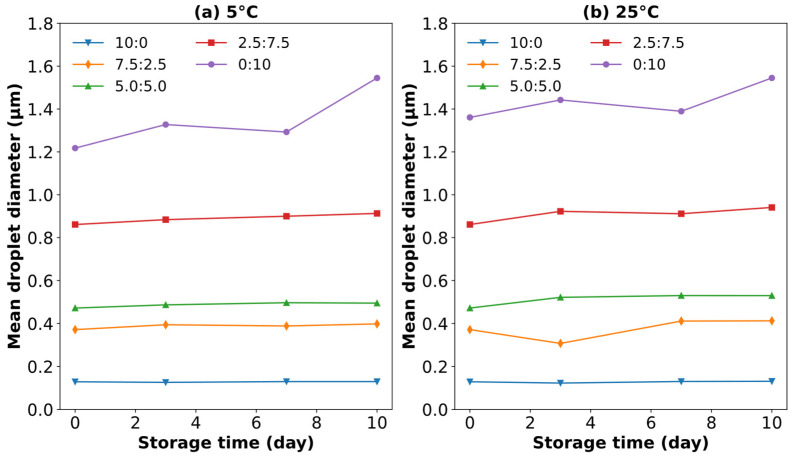
Mean droplet diameter (*d*_3,2_) of O/W emulsions stabilized by SCPs or *Quillaja* saponin at various weight ratios and stored for 10 days either at (**a**) 5 °C or (**b**) 25 °C (the weight ratios were 10:0, 7.5:2.5, 5:5, 2.5:7.5, and 0:10 (SCP: co-emulsifier)).

The emulsifying ability of SCPs combined with *Quillaja* saponins was evaluated using droplet size distribution and average droplet size analyses of the O/W emulsions at various weight ratios. Figure 11 shows the particle size distribution over a 10 d storage period at 5 °C and 25 °C, while Figure 12 shows the evolution of mean droplet diameter (*d*_3,2_) under the same conditions.

**Figure 13:**
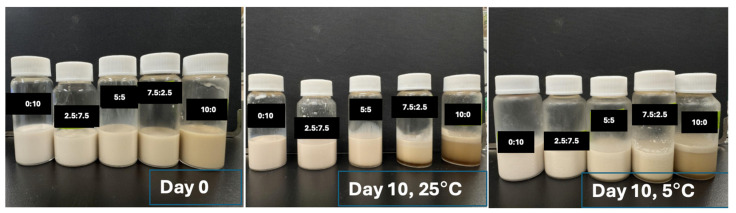
Emulsions stabilized by solubilized cassava peels loaded with varied ratios of *Quillaja* saponin as a co-emulsifier.

The results demonstrated a significant improvement in emulsion stability and uniformity compared to those of SCP alone. The mixture of SCPs to *Quillaja* saponins exhibited favorable outcomes with smaller average droplet sizes and more consistent distribution, especially at 5 °C storage conditions. These mixtures maintained smaller mean droplet diameters than SCP alone, resulting in better emulsions. The hypothesized synergistic effect between SCP carbohydrates and *Quillaja* saponins (Figure 13) can be explained by several mechanisms, such as complementary interfacial adsorption, where the amphiphilic nature of saponins, with their hydrophobic triterpene backbones and hydrophilic sugar moieties, allows them to adsorb at the oil–water interface [54]. Concurrently, solubilized carbohydrates from cassava peels, which likely contain both hydrophilic and hydrophobic regions, can be co-adsorbed at the interface. This co-adsorption creates a more densely packed and structurally diverse interfacial layer, enhancing the emulsion stability [48].

Another mechanism is the synergistic lowering of the interfacial tension. While saponins are known for their strong surface-active properties, the presence of SCP carbohydrates may further reduce interfacial tension. This synergistic effect can lead to smaller initial droplet sizes during emulsification and contribute to long-term stability [39].

Saponins are known for their stability across various pH levels, whereas carbohydrates from SCP may contribute additional charged groups, enhancing the electrostatic stabilization of emulsion droplets [24]. The synergistic interactions between the modified natural polysaccharides and commercial emulsifiers demonstrate the potential of these polymer systems to create stable colloidal dispersions. The molecular organization at the interface suggests that modified polysaccharide chains contribute to steric and electrostatic stabilization mechanisms.

This synergistic interaction between SCP carbohydrates and *Quillaja* saponins results in a more effective emulsifier system that creates and maintains a uniform distribution than SCP alone. The observed stability at different storage temperatures further supported the robustness of this combined emulsifier system and highlighted its potential for use in various food and pharmaceutical applications.

**Figure 14:**
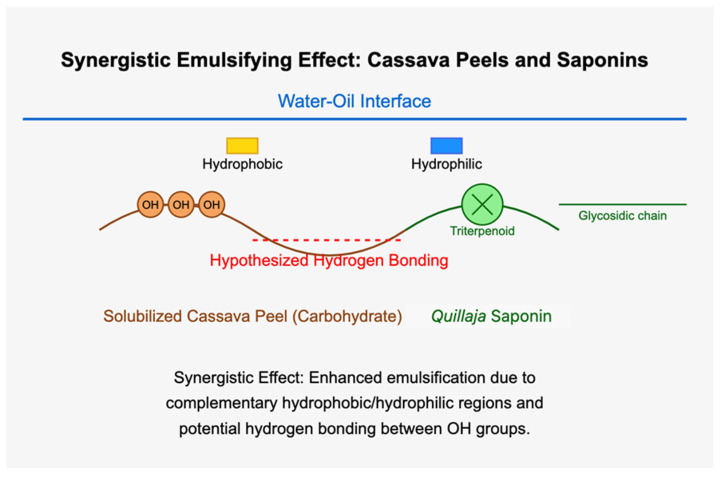
Schematic illustration of the potential synergistic interaction between SCPs and *Quillaja* saponins.

Figure 14 shows a hypothesized model of the enhanced emulsification mechanism of SCPs in combination with *Quillaja* saponins in an aqueous environment. This schematic diagram shows the potential synergistic interaction between these two amphiphilic compounds at the oil–water interface, although the oil phase is not explicitly shown in this water droplet model. The amphiphilic nature of the SCPs and *Quillaja* saponins is represented by their hydrophilic (blue circles) and hydrophobic (yellow squares) moieties, which are crucial for their emulsifier functions. The hypothesized bonding site, indicated by the red dashed line, suggests a possible hydrophobic interaction between the SCPs and *Quillaja* saponins, which could lead to the formation of a more robust interfacial film. This interaction resulted from complementary interfacial adsorption, enhanced steric stabilization, hydrogen bonding, and improved electrostatic repulsion between the emulsion droplets [54]. The orientation of these molecules at the interface, with hydrophilic regions extending into the aqueous phase and hydrophobic regions presumably oriented towards the oil phase, is consistent with the behavior of surface-active agents in emulsion systems [55]. This model provides a conceptual framework for understanding how the combination of SCPs and *Quillaja* saponins leads to smaller droplet sizes and enhanced emulsion stability, as observed in previous experimental studies.

Figure 14 shows the hypothesized synergistic emulsifying mechanism between SCPs and *Quillaja* saponins at the water–oil interface. SCPs, primarily composed of carbohydrates, have a polymeric structure with exposed hydroxyl (OH) groups, indicating their amphiphilic nature, which is consistent with the structural characteristics of cassava starch and fibers [56]. *Quillaja* saponins have a characteristic triterpenoid core representing the hydrophobic region and an extended glycosidic chain representing the hydrophilic portion [48]. These components interact at the water–oil interface, with their respective hydrophobic and hydrophilic regions oriented to minimize free energy, as described in classical emulsion theory [44]. A key feature of this interaction is the hypothesized hydrogen bonding between the cassava peel carbohydrates’ hydroxyl groups and the saponins’ glycosidic chains (indicated by the dashed red line). This molecular arrangement is proposed to enhance the emulsification efficacy through a complementary effect; cassava peel provides additional amphiphilic molecules to stabilize the interface, while saponins contribute to their strong surfactant properties [24]. This synergistic effect is hypothesized to improve the emulsion stability and reduce the interfacial tension, potentially leading to smaller droplet sizes and increased emulsion stability compared to either component alone. This phenomenon has been observed in other mixed emulsifier systems [57].

**Figure 15:**
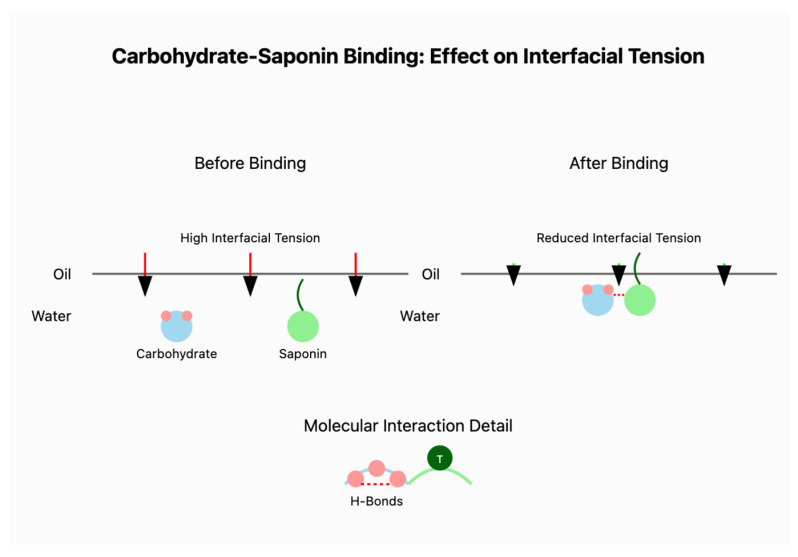
Schematic illustration of the carbohydrates–saponin binding mechanism that leads to a reduction in interfacial tension.

A schematic illustration (Figure 15) demonstrates the molecular mechanism underlying the synergistic interaction between solubilized cassava peel carbohydrates and saponins at the oil–water interface. Before binding, individual molecules exhibit limited surface activity, resulting in a higher interfacial tension. Upon interaction, hydrogen bonding occurs between the hydroxyl groups of cassava peel carbohydrates and the glycosidic chains of saponins. This molecular association facilitates the formation of a robust interfacial film, where the hydrophobic regions of the saponins (triterpenoid core) are oriented toward the oil phase, whereas the hydrophilic portions of both molecules extend into the aqueous phase. This complementary arrangement leads to enhanced surface coverage and steric stabilization, reducing the interfacial tension. This stabilization mechanism was further supported by decreased droplet size distribution and enhanced emulsion stability over extended storage periods. The molecular organization at the interface suggests that the carbohydrate–saponin complexes create a more densely packed interfacial layer than either component alone, contributing to the observed synergistic enhancement of the emulsifying properties.

## 4. Conclusions

This study demonstrates the potential use of SCPs, derived through upcycling underutilized cassava peels as a co-emulsifier when combined with *Quillaja* saponins and Tween 20. The observed synergistic effects at concentration ratios through 7.5:2.5 to 2.5:7.5, manifested through reduced interfacial tension and modulated zeta potential, represent a notable advancement in emulsion technology. This study advances the understanding of natural polysaccharide modification through controlled hydrothermal processing, demonstrating how agricultural waste can be transformed into functional polymeric materials. The insights gained regarding structure–property relationships in these modified natural polymers contribute to the development of sustainable, bio-based alternatives to synthetic materials. Importantly, SCPs showed efficacy as co-emulsifiers under milder processing conditions (127 °C at 0.2 MPa) compared to more intense conditions (140 °C at 2 MPa) suggesting that energy-efficient methods can be employed without compromising the functional properties. Despite the cheap cost of cassava peels, further studies need to be carried out to understand cost analysis and the economic impact for application on an industrial scale. The different behavior of SCPs with *Quillaja* saponins versus Tween 20 provides valuable flexibility in formulation design, while contributing to the growing trend toward green chemistry in food and cosmetic applications.

This study was limited to specific concentration ratios and processing conditions, and the long-term stability of the emulsions beyond 10 days was not evaluated. Additionally, the impact of seasonal variations and age on the SCP functionality of cassava peel was not assessed, and the potential presence of antinutritional factors in the modified SCPs was not investigated.

While these findings demonstrate the potential of valorizing agricultural byproducts in sustainable emulsion systems, future research should focus on understanding the underlying molecular mechanisms, evaluating performance in natural food systems, and assessing the economic viability of industrial-scale production. Future studies should investigate the molecular interactions between SCPs and different emulsifiers, optimize the processing conditions for industrial scale-up, and evaluate the impact of storage conditions on emulsion stability. Research should also examine potential applications in different food matrices, assess the environmental impact through life cycle analysis, and develop standardized quality control methods for commercial production. Comprehensive safety assessments and regulatory compliance studies are required before commercial implementation.

This study represents a significant step toward developing more sustainable and functional emulsion systems with potential impacts across multiple industries while emphasizing the value of agricultural byproducts as sources of functional ingredients.

## Figures and Tables

**Table 1:** The major composition of untreated cassava peels and the major composition of solubilized cassava peels (SCPs) obtained by autoclave treatment at 127 °C at 0.2 MPa.

**Compo** **und**	**Untreated Cassava Peels**	Solubilized Cassava Peels
Total carbohydrates	38.5 wt%	3.34 wt%
Ash	8.6 wt%	3.89 wt%
Total protein	9.31 wt%	0.765 wt%
Cyanide	0.00065 wt%	0.000433 wt%

**Table 2:** Interfacial tension and zeta potential of solubilized cassava peels (SCPs) treated using HTS or autoclave.

Sample	Operating Conditions	Interfacial Tension (mN/m) *	Zeta Potential (mV)
HTS-treated SCPs	140 °C; 2 MPa; 15 min	12.94	−3.09
Autoclave-treated SCPs	127 °C; 0.2 MPa; 15 min	13.14	−8.23
Water (reference)	--	25.0	-

* Interfacial tension values reported in the table were measured for each sample and soybean oil.

## Data Availability

All supporting data are contained within the manuscript.
